# COVID-19 school closures, learning losses and intergenerational mobility

**DOI:** 10.1057/s41599-026-06967-w

**Published:** 2026-03-21

**Authors:** Alexandru Cojocaru, Joao Pedro Azevedo, Ambar Narayan, Veronica Montalva Talledo

**Affiliations:** 1 World Bank, Washington D.C., USA; 2 United Nations Children’s Fund, New York, NY USA

**Keywords:** Development studies, Economics

## Abstract

The paper investigates the longer-term inequality implications of COVID-19 by examining the ability of children from different backgrounds to engage in continued learning throughout the pandemic, and their implications for intergenerational mobility in education. The analysis builds on data from the Global Database of Intergenerational Mobility, country-specific learning loss simulation models using weekly school closure information from February 2020 to February 2022, and high-frequency phone survey data collected by the World Bank during the pandemic to assess the incidence and type of continued learning during school closures across children from different backgrounds. Using this information, the paper simulates counterfactual educational attainment and corresponding absolute and relative intergenerational educational mobility measures to estimate COVID-19 impacts. The results suggest that the extensive school closures are likely to have a significant impact on both absolute and relative mobility in the absence of remedial measures – in upper-middle-income countries, the share of children with more years of education than their parents could decline by 8 percentage points. Unequal access to continued learning across children from different socioeconomic backgrounds also leads to a significant decline in relative mobility. Notably, adopting more optimistic assumptions about the effectiveness of alternative learning modalities makes the simulated decline in relative mobility even larger.

## Introduction

The COVID-19 pandemic has had a devastating impact on poverty and well-being worldwide, and this impact has not been equally distributed across or within countries. Those with per-capita incomes between $1.9/day and $5.5/day experienced the largest income drops globally (Narayan et al. 2022). Data from High Frequency Phone Surveys (HFPS) collected by the World Bank throughout the pandemic show higher rates of work stoppages and income losses in low- and middle-income countries compared to high-income countries (Bundervoet et al. 2023; Narayan et al. 2022). Ferreira et al. (2021) show that the number of years in poverty resulting from the pandemic is inversely correlated with the country’s GDP per capita. Those with lower levels of education or more precarious connections to the labor market were more severely affected by the restrictions to economic activity (Brunckhorst et al. 2023a; Hill and Narayan 2020).

While the patterns of pandemic impacts appear to have been dis-equalizing, estimates of short-run changes in income inequality due to COVID-19 tend to be small in magnitude, and short-term increases in inequality during the 2020–2021 are not universal (Narayan et al. 2022; World Bank 2022a). There is a concern, however, that the inequality impacts of the pandemic will be amplified over the medium to long-term by learning losses due to school closures. Access to continued learning during school closures has been strongly and positively correlated with GDP per capita across countries, and with the level of parental education within countries (Narayan et al. 2022; Josephson et al. 2020). This can lead to a highly unequal pattern of losses in human capital formation across socio-economic groups, whose impacts are likely to last through the life-cycle.

The main goal of this paper is to explore these medium-to-long-term inequality impacts COVID-19 through the education channel. Specifically, we study inequality traps by estimating the impact learning losses associated with school closures on inter-generational mobility in education. Following Narayan et al. (2018), we consider two mobility concepts: *absolute mobility* that measures the share of individuals who surpass the education of their parents, and *relative mobility* that captures the degree to which individual socioeconomic outcomes are independent of the outcomes of one’s parents (van der Weide et al. 2021). These indicators reflect two broad concepts of societal well-being: a universal human aspiration of parents hoping for a better life for their children, and inequality of opportunity in a society (Narayan et al. 2018; Corak 2013). Unlike the well-documented impacts of the pandemic on monetary well-being (World Bank 2022b), this study focuses on one of the key non-monetary indicators of quality of life, namely on educational attainment and its linkages with economic inequality.

While a number of studies have examined the implications of school closures on learning outcomes and dropout rates (see Moscoviz and Evans 2022; Patrinos et al. 2022; Jakubowski et al. 2023; Patrinos 2023; Schady et al. 2023; Jakubowski et al. 2024; Dela Cruz et al. 2025), we are only aware of two studies that examine the impact of school closures on inter-generational mobility for a set of 17 Latin American countries (Neidhofer et al. 2021), and, using a similar methodology, for a sample of 8 countries in Sub-Saharan Africa (Neidhofer et al. 2022). The contribution of our study is thus two-fold. First, we estimate the impacts of school closures on educational persistence for a larger set of developing countries across several regions of the world. Second, unlike earlier studies, which draw on pre-pandemic data and require assumptions on pandemic learning modalities across households, we are able to observe the types of learning (if any) that children from different types of households have engaged with during school closures. This allows for fewer assumptions to simulate the distributional gradient in learning losses within each country.

The key findings of the simulations indicate that the COVID-19-induced widespread school closures can result in significant reductions in absolute and relative educational mobility across generations. The average proportion of children exceeding the education of their parents could decrease by 8–9 percentage points in high- and upper-middle-income countries. The higher incidence of disengagement among children from households with lower socio-economic status during school closures may have resulted, depending on assumptions regarding the effectiveness of pandemic learning, in an almost 4% decline in relative intergenerational mobility, measured by the correlation coefficient between parents’ and children’s education. These effects are significant given the evolution of relative mobility across birth cohorts from 1950 to 1980.

The findings of the simulations in this study are best seen as illustrative and reflecting what might happen under model assumptions, rather than being a statistical prediction of the future. The qualitative interpretation of the results in terms of their relative magnitudes compared to historical trends is thus more instructive than the precise numbers generated by the simulations. The latter could also change with new data or data for missing countries becoming available. The discussions and limitations section reviews the implications of the key assumptions of the model and the biases they may introduce into the interpretation of the results.

The rest of the paper is structured as follows. Section “Context and motivation” provides the context and rationale for the choice of intergenerational mobility in education as the outcome of interest and discusses how the paper adds to the literature on longer-term socioeconomic impacts of the pandemic. Section “Data and methods” describes the data employed in the analysis and the empirical methodology. Section “Main results” presents an overview of the key simulation results and sensitivity analysis. Section “Discussion and limitatio**ns**” provides a discussion of the results and some of the methodological limitations. Further details related to learning loss simulations are available in the Online Appendix.

## Context and motivation

This study aims to contribute to the literature on long-term impacts of the pandemic on societal well-being by providing evidence on how disruptions in schooling and work experience can widen existing inequalities within and across countries. Earlier studies have been limited, by design or data availability, to simulating aggregate impacts on human capital and productivity, namely losses in global learning and output (Azevedo et al. 2021; Fuchs-Schundeln 2022; Samaniego et al. 2022), or the implications of loss-of-learning effects on labor productivity and the speed of economic recovery (Buffie et al. 2022).

This study examines how the unequal effects of learning disruptions among socioeconomic groups within countries influence social mobility, and is most closely related to that of Neidhofer et al. (2021) and Neidhofer et al. (2022). Unlike in previous studies, that rely on assumptions about the differential ability of parents with high or low levels of education to substitute for school instruction, we are able to observe directly whether, and by what modality, children in particular households were engaged in learning during periods of school closures, drawing on evidence that school closures disproportionately affected children from poorer families, particularly in low-income countries, who had little or no access to learning opportunities (Bundervoet et al. 2023, World Bank 2023). This heterogeneity in pandemic learning can lead to disparities in learning losses across and within countries and reduce socioeconomic mobility of the current generation of children, thereby exacerbating inequality over the long term.

The study focuses on mobility in education for two main reasons. First, human capital development is a key predictor of lifetime earnings, and relative mobility in education is a good proxy for relative income mobility (Narayan et al. 2018). Second, estimation of educational mobility involves fewer methodological and data challenges compared to income mobility. Education, once acquired, does not vary across the life cycle, and intergenerational data on education is much more widely available and much less noisy in the typical recall-based income measures.^1^

Any simulations of pandemic impacts on future educational or income outcomes assume that human capital impacts of large shocks persist over time. Empirical evidence from past disasters supports this assumption. Disrupted schooling and the trauma of shocks can adversely impact academic performance and produce differences that are observable years later (Gibbs et al. 2019). The Zimbabwe drought of 1982–84 resulted in a delay in starting school of 3.7 months and 0.4 grade less of completed schooling, which led to a 14% reduction in lifetime earnings (Alderman et al. 2006). The 1916 polio pandemic in the United States resulted in young people aged 14–17 having lower educational attainment compared to slightly older peers (Meyers and Thomasson 2017). Four years after the 2005 earthquake in Pakistan, even though households near the fault line were similar in terms of monetary well-being to households living further away, children living within 10 kilometers of the fault line had significantly lower test scores (Andrabi et al. 2021). The World Bank estimates that globally, pandemic-related learning losses could lead to between US$23,514 and US$31,800 in lost earnings over a typical student’s lifetime (Schady et al. 2023).

## Data and methods

Estimating the impact of the COVID-19 pandemic on intergenerational mobility ideally requires three main ingredients:

(i) A baseline measure of educational attainment (in years of education) and inter-generational mobility for the current cohort of students in the absence of the COVID-19 shock.
(ii) A way of translating COVID-induced learning losses into losses in educational attainment in years of education, which underlie mobility measures.
(iii) Data that describe the heterogeneity of learning experiences across different households.

### Baseline educational attainment and mobility estimates

For baseline estimates of inter-generational mobility, we rely on data from the Global Database on Intergenerational Mobility (GDIM), which contains estimates of absolute and relative intergenerational mobility (IGM) by 10-year cohorts, covering individuals born between 1940 and 1989 (GDIM 2020). Following Neidhofer et al. (2021) and Neidhofer, Lustig and Larroulet (2022), we rely on mobility estimates from a cohort of children who are closest in age to the children who were in school in 2020-21, when school closures took place, namely children born between 1980 and 1989, most of whom would have completed their secondary education during the 2000s.^2^

**Fig. 1:**
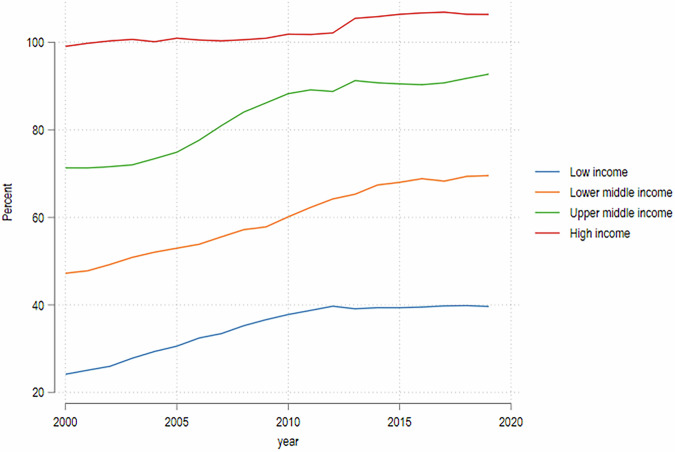
Secondary enrollment rates (% of gross). World Development Indicators, World Bank.

How consequential are the data constraints that prevent us from working with a more recent birth cohort? Even though the current school-going cohort (born during 2000–2015) would have had better education outcomes than the 1980s cohort in the baseline case (absence of the pandemic) in most developing economies, our analysis is not focused on the absolute changes in educational attainment, but rather on the difference between two scenarios represented by two vectors of educational attainment generated for the same cohort of individuals – one without COVID-19 (baseline) and the other accounting for the impacts of the pandemic (counterfactual). Any secular trend in educational attainment from the 1980s cohort to the current school-going cohort would affect both scenarios equally and have no effect on the impact of the pandemic obtained from the difference between these scenarios. Moreover, even though there is a secular time trend in educational attainment for most developing economies, the rate of increase appears to have slowed down over time, with enrollments flattening out across all income groups of countries over the period 2011–2019 (Fig. 1).

One may worry, nonetheless, that the secular increases in educational attainment between the 1980s and 2000s birth cohorts occurred at differential rates between the corresponding cohorts of parents and children (as may be the case with the flattening gradient of educational attainment growth), or that educational attainment increased at different rates between children from different socio-economic backgrounds. The potential biases of these types of dynamics are discussed in more details in Section “Discussion and limitations”.

The GDIM covers 153 countries, which are home to 96 percent of the world’s population, allowing us to provide the first global picture of COVID-19 impacts on educational mobility. We use several measures of inter-generational mobility in the GDIM that have been analyzed extensively in Narayan et al. (2018) and van der Weide et al. (2021). The first measure is *absolute educational mobility*, measured by the share of a generation in a country who have greater educational attainment (such as years of schooling) than the highest educational attainment among (both) their parents, conditional on parents not having the maximum years of schooling observed in the sample, so that everyone has a chance of surpassing their parents. The second measure is *relative educational mobility*, which measures the extent to which an individual’s position in the distribution of educational attainment, measured in terms of years of schooling, is independent of the position of his or her parents in their distribution of educational attainment, in terms of their correlation coefficient. A lower correlation between children’s and parents’ years of schooling is indicative of higher relative mobility (or lower educational persistence).

### Learning losses associated with COVID-19 and educational attainment

The learning loss simulations estimate how COVID-driven school closures affected Learning Adjusted Years of Schooling (LAYS) in 2022, based on a concept that combines quantity (access) and quality (learning outcomes) of schooling into a single metric of progress (Filmer et al. 2020). By capturing the educational life of students from 4 to 17 years, it encompasses all levels of basic education. We rely on the simulation results from Azevedo et al. (2022) to shock the expected learning of the current cohort of the student population. The main parameters of the simulation model are outlined in Annex 1.

To simulate intergenerational mobility, we need to impute losses in LAYS into simulated counterfactual educational attainment in terms of years of schooling in the GDIM. We build on the relationship between expected years of schooling, learning-adjusted years of schooling and self-reported educational attainment outlined in Annex 2, to pass-through the simulated learning losses in LAYS to attainment. First, we calculate the shares of LAYS lost due to COVID-19 in % terms of pre-pandemic value. A student expected to complete 10 years of schooling adjusted for learning and forecast to lose 1 year of LAYS due to COVID-19, will lose a *delta*_*lays*_ = 1−[(10−1)/10] = 0.1 or 10% share of schooling. We then compute the average educational attainment for the 1980s cohort for each country, and the equivalent absolute loss using *delta*_*lays*_. For example, if the expected years of schooling from GDIM is 8 years on average, the equivalent COVID-19-induced loss would be 0.8 years (10%). Thus, we subtract 0.8 years from the registered educational attainment for all students in that country.

As in Neidhofer et al. (2021) and Neidhofer et al. (2022), the simulations are in the absence of learning recovery and acceleration in the post-pandemic years. In part, this is to inform the debate around the potential long-term consequences of this shock for this generation of students and the associated cost of inaction, and in part because we do not observe the learning recovery response in developing countries, albeit emerging evidence suggests that learning recovery will be challenging, even in high-income countries (Kuhfeld and Lewis (2022); Angrist et al. 2021) and especially in low-income countries (Schady et al. 2023). To conserve space, more details on the available evidence related to recovery and acceleration is presented in Annex 1.

In light of these methodological considerations, the simulated measures of post-pandemic educational attainment should not be interpreted as a prediction of what educational attainment of countries will look like after the pandemic. Rather, we are using the LAYS framework to shock the expected years of schooling by the length of the school closure and further adjust this value by the lower expected learning in Harmonized Test Scores (HTS) points. This also accounts for the potential income effect on student enrollment, but this last mechanism seems to be of negligible magnitude (Annex 2).

### Constructing a distributional gradient consistent with national LAYS estimates

**Fig. 2:**
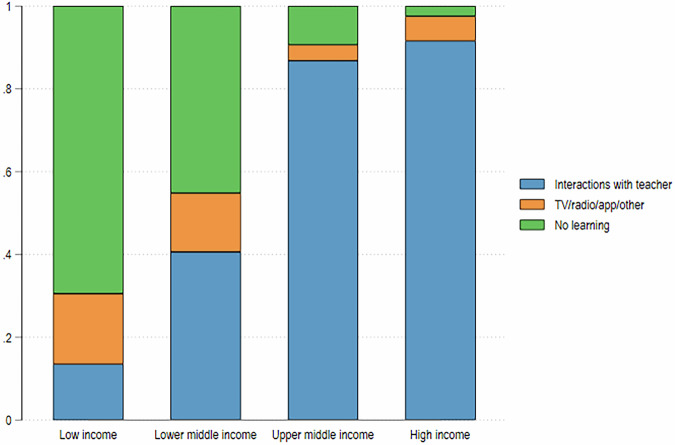
Composition of learning modalities during school closures by country income group. Authors’ estimates based on HFPS data from a sample of 30 developing countries, including 5 LICs, 11 LMICs, 12 UMICs and 2 HICs.

To construct distributionally sensitive simulations of COVID-19 impacts, we rely on information from HFPS data on the learning experience of children during school closures. A key advantage of HFPS data is that it provides, for a sample of 30 countries, parent-reported information of whether, and how, a particular child was learning during school closures, differentiating between (i) completing assignments provided by the student’s teacher; (ii) other remote learning modalities not involving direct interactions with the student’s teacher, such as watching educational TV programs, listening to educational programs on the radio, using mobile learning apps, or other similar modalities. To our knowledge, this is the only source of comparable household-level data on actual learning engagement of children throughout school closures for a large set of developing countries. The data show that in upper-middle and high-income countries students for the most part were able to maintain interactions with their teachers, while in low and lower-middle-income countries a large share of students were not learning at all, and among those who were learning, remote learning modalities that do not involve direct interactions with the students’ regular teachers, such as learning through TV, radio, or mobile apps was considerably more prevalent (Fig. 2).

**Fig. 3:**
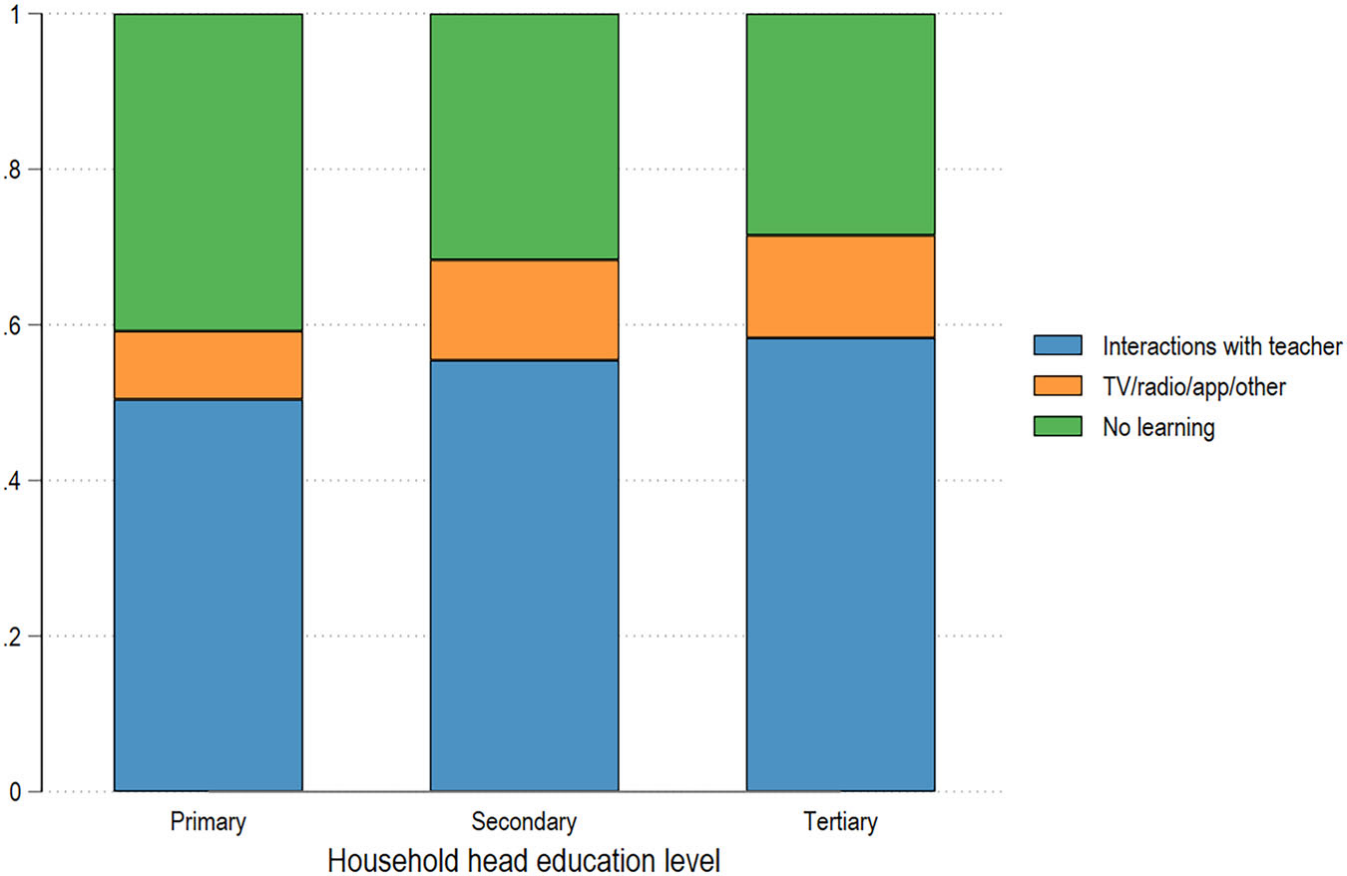
Distribution of learning modalities during school closures, by level of parental education. Authors’ estimates based on HFPS data from a sample of 30 developing countries, including 5 LICs, 11 LMICs, 12 UMICs and 2 HICs.

A second advantage is that learning information can be paired with parental education, to estimate how learning losses were distributed between different socioeconomic groups within a country. While the extent of learning loss for each child must still be estimated using assumptions, the estimate is informed, crucially, by the observed type of learning engagement that the child participated in.^3^ Given the focus on inter-generational mobility, we assess how learning engagement of students during school closures varies by differences in parental educational attainment (whether primary, secondary, or tertiary). The share of children who are not learning at all decreases monotonically with the level of education of parents, while the share of students who had interactions with teachers increases with parental education (Fig. 3).

HFPS data has several limitations that the reader should be aware of, related to over-representation of household heads in countries where the sample was based on a pre-pandemic household survey, and based on the phone-owning population in countries where data was collected by Random Digit Dialing (RDD). Sampling weights were constructed to adjust for differential response rates among population subgroups, to approximate nationally representative estimates. We employ solely the information related to differences in learning modalities of children by parental education, which is less likely to be affected by concerns about representativeness of the sample, albeit HFPS data appear to be well-behaved overall (Kugler et al. 2023; Brunckhorst et al. 2023b).

Since we do not directly observe learning losses for each student from the LAYS simulations in Azevedo et al. (2022), we need additional assumptions to undertake distributionally sensitive simulations. Analytically, the relative changes of LAYS depend on a number of factors like the length of school closures, the extent of the income shock, the elasticity of dropout to income, the effectiveness of mitigation efforts in the country (which depends, for instance, on the coverage of remote learning measures deployed by governments). Children’s engagement in learning activities, which we observe, is an outcome of all these factors. For example, a child may not engage in any learning activity because there is no coverage of remote learning where she lives, or because her family lost income and she needs to work.

To make progress, we assign a “loss index” to each type of learning activity, calculate the average learning loss index for children in each parental education group, and assume that the variation in the loss of GDIM years of schooling by parental education group is proportional to the variation in the average learning loss score by parental education group. This proportionality assumption is defensible because the HFPS information on children’s engagement in learning activities and the relative changes of LAYS are both realizations of the effects of a similar set of factors that influence learning outcomes. In order to translate the pandemic learning experience (or lack thereof) into learning losses, we assume that for children who are not learning, the loss, expressed in years, is equivalent to the share of a given academic year that the school was closed. If one imagines a loss index that ranges from 0 for normal pre-pandemic schooling to 1 in the case of no schoolwork of any kind, the children who report that they were not learning by any means during school closures are assigned a loss score of 1, while students who were learning by way of various means during the pandemic will have a loss index somewhere above 0 to capture some loss of learning effectiveness during the pandemic but below 1, as pandemic learning would have had some, even if limited, effectiveness.

While it is plausible to assume, in an ordinal sense, that in person interactions with the teacher may be more effective – and thus generate a lower learning loss – than learning through TV/radio, and the latter, in turn would generate a lower loss than no learning at all during school closures, we have no way of validating the cardinal measures of these loss magnitudes. In the baseline scenario 1, we rely on the evidence of low effectiveness and poor take-up of alternative learning modalities (Schady et al. 2023) to assign loss index values of {0.3; 0.8; 1}, while in scenarios 2–4 we assign several alternative values to the loss index for each learning modality to investigate the sensitivity of the results to alternative assumptions, varying one component at a time (Table 1).

**Table 1:** Loss index values across simulation scenarios.

Scenario	Direct interactions with teachers	TV/radio/app/other	No remote learning
1	0.3	0.8	1
2	0.1	0.8	1
3	0.1	0.6	1
4	0.1	0.3	1

The loss indices assigned to each learning modality do not have an intrinsic socio-economic gradient. A child from any household who is engaged in learning by way of direct interactions with their teachers during the period of school closures is assigned the same loss index. However, the incidence of learning modalities is not uniform across population groups. Students whose parents have lower levels of education tend to have a higher loss index on average because they are more likely to report no learning during school closures, or less effective learning modalities, whereas students with better-educated parents have a higher prevalence of learning based on direct interactions or completing assignments from their teachers.

To make the distributionally sensitive simulations internally consistent with the national LAYS estimates, we impose a constraint that the learning losses associated with the different triplets of loss index values are such that, given the observed distribution of children across the different learning modalities and the loss index associated with each of these learning modalities, the average national loss matches the national average loss in years of schooling for each country in our sample. To do so, we express the national average loss as follows:

1 $${\delta }_{i}=\mathop{\sum }\limits_{j}{\delta }_{{ij}}* {{share}}_{{ij}}$$

where ${\delta }_{i}$ is the average national learning loss in years in country *i*, ${\delta }_{{ij}}$ is the average learning loss in country *i* for children with parental education *j* and ${{share}}_{{ij}}$ is the share of children with parental education *j* in that country. Simulations with uniform losses are a special case of (1) where the loss ${\delta }_{{ij}}$ is the same for all children in country *i*. The distributionally sensitive simulations relax this assumption, but note that we do not observe the ${\delta }_{{ij}}$ values for each of the three parental education groups. We recover them, given our assumptions on the learning loss index as follows:

2 $${\delta }_{{ij}}={x}_{i}* {{LI}}_{{ij}}$$

where *x*_*i*_ is an unknown parameter, and ${{LI}}_{{ij}}={\sum }_{k}{{LI}}_{k}* {{share}}_{{jk}}$ is the average learning loss index for children with parental education *j*, computed as a weighted average of the learning efficiency of each education modality *k* multiplied by the share of children with parental education *j* who are engaged in that learning modality. Substituting into (1) and solving for *x*_*i*_, provides us, for each country *i* with unique values of ${\delta }_{{ij}}$ that sum up to the national estimate of the learning losses ${\delta }_{i}$, given the weights associated with the educational distribution in that country.

## Main results

We start with the benchmark case in which the average loss in learning for each country is applied uniformly to all children in that country. While not realistic, it allows us to highlight what is lost by ignoring the differential learning experience of children from different socio-economic groups. This is particularly relevant for the case of relative intergenerational mobility, because assuming uniform losses in years of education for all children in a country would leave measures of relative mobility unchanged.^4^ Thus, any observed changes in relative mobility are attributable to the within-country heterogeneity of educational outcomes.

**Fig. 4:**
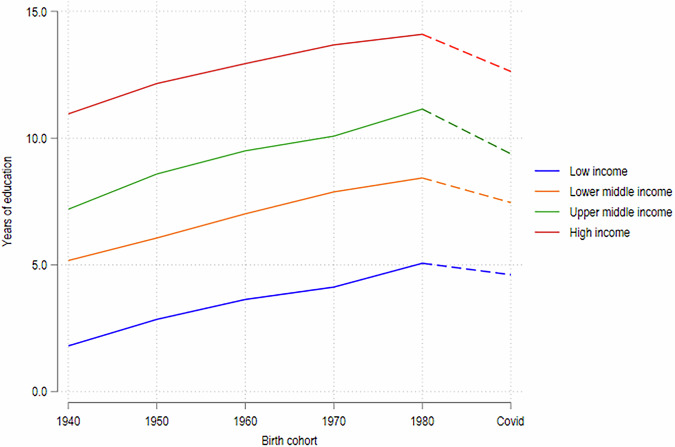
Evolution of educational attainment over time and based on the COVID-19 scenario. Authors’ estimates based on data from Azevedo et al. (2022) and GDIM. Estimates based on the years of education of the 1980s cohort, and thus rely on the assumption that the years of education of the 1980s cohort roughly reflect the years of education for the current school-going cohort (roughly 2000–2015). Estimates in Fig. 1 suggest that this is not a very strong assumption.

We estimate that children’s educational attainment could fall by more than a full year (1.2 years overall) on average. This impact is more pronounced in middle-income countries, whereas it is relatively smaller in low- and high-income countries, reflecting the heterogeneity in the extent of school closures (Annex 1). To understand the magnitude of the implied changes in educational attainment, it helps to examine them against their historic evolution (Fig. 4). For upper-middle- and high-income countries, the simulated loss in educational attainment induced by the pandemic would be equivalent to reversing the progress made by the 1970s and 1980s cohorts compared to the 1960s cohort. For lower-middle and low-income countries the impact is less pronounced, but even for low-income countries the loss in educational attainment is equivalent to reversing most of the progress made by the 1980s cohort over the 1970s cohort.

**Fig. 5:**
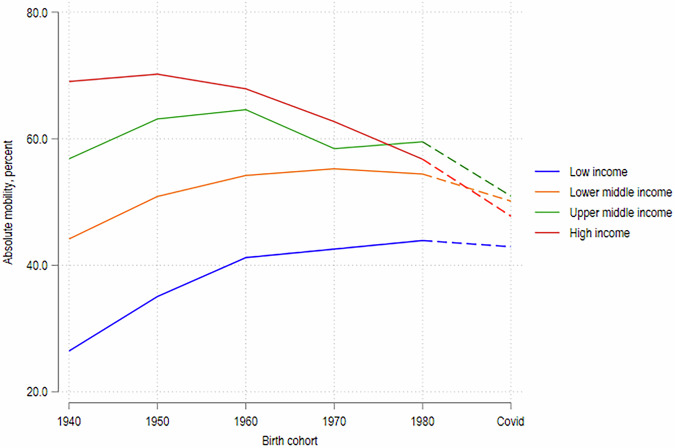
Evolution of absolute mobility by birth cohort. Authors’ estimates based on data from Azevedo et al. (2022) and GDIM.

The largest decreases in absolute intergenerational mobility in education are similarly observed in high- and upper-middle-income countries, where the share of children with more years of education than their parents is estimated to decrease by 9 percentage points and by 8 percentage points, respectively, whereas in lower-middle-income countries the change is only 4 percentage points and smaller still in low-income countries. The simulations imply that the impact of the COVID-19 learning losses would reverse the past improvements in absolute mobility in low- and lower-middle-income countries, while exacerbating the recent decline in absolute mobility in upper-middle and high-income countries (Fig. 5). As stated earlier, these simulations assume that absolute mobility for the current school-going cohort (born, approximately, during 2002–2015) in the absence of COVID-19 would have been similar to what was observed for the cohort born in the 1980s, which we find to be plausible on the basis of pre-existing trends: absolute mobility was almost flat (in LICs and LMICs) or declining (in UMICs and HICs) between the cohorts of the 1970s and 1980s, and secondary enrollment rate changed very little for all groups of countries since 2011 (Fig. 1). In Section “Discussion and limitations” we discuss alternative assumptions on the baseline mobility of the current birth cohort and their implications.

With these benchmark results in mind, we proceed with simulations that capture the differential effectiveness of different learning modalities, and differences in the use of each modality across population groups. These simulations indeed show a strong socio-economic gradient in the magnitude of learning losses. For instance, while the national mean absolute loss in years of education in our sample is 1.4 years, this varies from 1.5 years for children whose parents have less than primary education to 1.3 years for children with parents who have tertiary education – a 13 percent difference between the top and bottom parental educational categories. The difference in relative losses is even greater, given the much lower average years of schooling attained by these two groups of children when they reach adulthood (6.9 years of education and 13.3 years of education, respectively). For those children whose parents have at most pre-primary education (ISCED0), the COVID-19-related loss in educational attainment amounts to 23 percent of the no-COVID baseline, compared to 10 percent among those whose parents have tertiary education (ISCED5).^5^

**Fig. 6:**
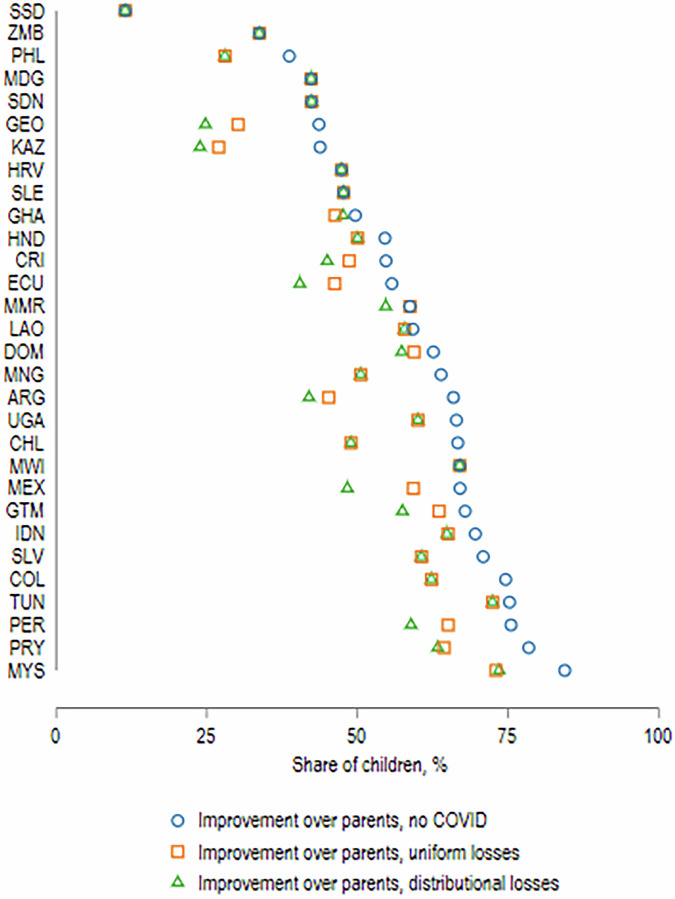
Share of children with improved education over their parents under different scenarios. Authors’ estimates based on data from Azevedo et al. (2022) and GDIM. Distributional losses based on Scenario 1 assumptions on the loss index. These results are not affected by alternative loss index assumptions for the vast majority of countries, with the exception of Guatemala and Chile, where absolute mobility declines further in alternative scenarios. We thus omit alternative scenarios from the graph for ease of legibility.

How do these losses of educational attainment translate into changes in absolute and relative inter-generational educational mobility? Fig. 6 plots, for countries in our HFPS sample, the share of children who have more years of education than their parents for three cases: (i) without the impacts of COVID-19 school closures, (ii) accounting for COVID-19 with uniform losses; and (iii) accounting for COVID-19 with distributionally sensitive losses. The estimates suggest that relaxing the assumption of uniform losses within countries worsens the decline in absolute mobility in a number of countries (e.g.: Argentina, Costa Rica, Georgia, Myanmar). At the same time, in most countries the main loss in absolute mobility vis-à-vis no COVID case is on account of the overall loss in educational attainment, rather than the differential impacts across socio-economic groups. Given the restriction that differential losses across population groups add up to the average national loss, based on educational and learning modality composition, it is not surprising that the uniform loss simulations capture a large share of the impact of learning losses on absolute intergenerational mobility.

**Fig. 7:**
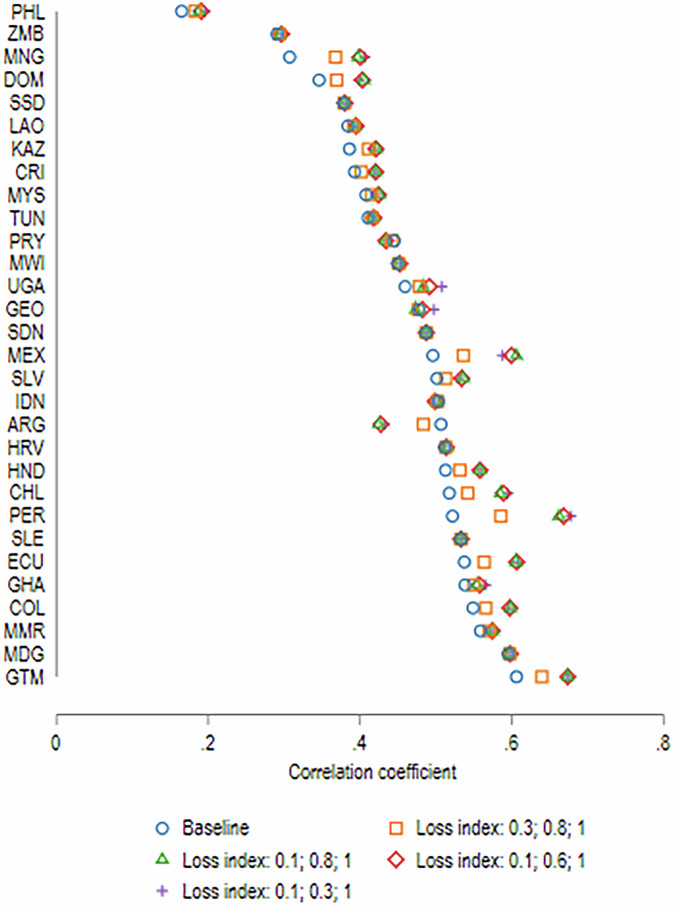
Relative mobility (correlation coefficient) under alternative loss index scenarios. Authors’ estimates based on data from Azevedo et al. (2022) and GDIM.

Next, we examine the impact of relaxing the assumption of uniform losses on our measure of relative mobility (Fig. 7). Recall that uniform losses in years of education have no effect on measures of relative mobility as they do not affect the slope of the regression of children’s education on parental education. This implies that the changes in relative mobility vis-à-vis no-COVID are entirely attributable to differential losses in learning *within each country* resulting from (a) the differences in effectiveness of different learning modalities, and (b) the differential incidence of these modalities across children with different levels of parental education, which is also a sound proxy for socio-economic status.

Taking the values of the loss index under scenario 1, the intergenerational persistence between parents and children’s educational attainment increases by almost 4 percent. In several countries, the decline in relative mobility is much higher – 13 percent in Peru, 19 percent in Mongolia, 9 percent in Mexico and 8 percent in Philippines. Argentina is the only country in our sample where the correlation between parents and children’s education declines slightly in the distribution-sensitive scenarios.^6^ The estimates from the HFPS sample of countries thus suggest that abstracting from the within-country inequalities in children’s ability to engage in effective learning during the periods of school closures leads us to underestimate the impact of COVID-19 on inter-generational mobility.

The magnitudes of the decline in relative mobility are consistent with those previously estimated for Latin American countries (Neidhofer et al. 2021). They are also large relative to the historic evolution of relative mobility. van der Weide et al. (2021) show that relative mobility has been stable in high-income economies for those born in the 1950s–1970s, and increased by over 2 percentage points for the youngest 1980s cohort, having fallen by about 9 percent between 1960s and 1980s cohorts in developing countries. Given the slow evolution of mobility measures across 10-year birth cohorts, the immediate impacts of COVID-19 are meaningful; in our sample the average decline in relative mobility is equivalent to about half of the improvement of relative mobility observed in developing countries between the 1960s and 1980s cohort.

If we adopt more optimistic assumptions with respect to the effectiveness of learning during school closures (scenarios 2–4 described in Section “Constructing a distributional gradient consistent with national LAYS estimates”), the degree of intergenerational persistence would be exacerbated even further for many countries, particularly in the Latin America region. In other words, greater optimism about the effectiveness of alternative pandemic learning modalities (among those engaged in learning during school closures), implies a higher simulated loss of relative mobility, on average. This a priori non-intuitive result is due to the fact that children from households with lower parental education were relatively more likely to be completely disengaged from learning during the pandemic. As such, more optimistic assumptions vis-à-vis the effectiveness of pandemic learning would advantage children from higher socio-economic groups more.

## Discussion and limitations

The simulations suggest that the pandemic-induced learning losses are likely to have a significant impact on intergenerational educational mobility. This is a direct result of the uneven distribution of learning losses, in both absolute and relative terms within countries. Children with parents who have less than primary education are, under certain assumptions, estimated to experience almost double the average absolute loss in years of education relative to pre-pandemic levels compared to children of parents with tertiary education, because of the differences in the extent and type of learning during school closures. Notably, the uneven distribution of relative losses within countries would have occurred even if aggregate losses of a country were uniformly distributed, just because the same absolute loss is much larger in relative terms among those with lower levels of expected educational attainment. On the whole, education disruptions due to COVID-19 are likely to have worsened pre-existing educational inequality between socio-economic groups in most countries, increasing inequality of opportunity in the society.

The effects on intergenerational mobility are significant. The decline in absolute mobility that is attributable to the pandemic is particularly strong for high and upper-middle-income countries in our sample, reinforcing their documented declining trend in absolute mobility between the cohorts of the 1970s and 1980s. While a smaller decline in absolute mobility due to the pandemic is estimated for low and lower-middle-income countries, the impacts remain concerning. Absolute mobility in these countries remains lower than in high and upper-middle-income countries, despite much lower levels of educational attainment that leave plenty of scope for children to exceed the education levels of their parents. To a large extent, the simulations reveal that impacts on absolute mobility are driven by the extent of aggregate learning losses in each country rather than the distribution of learning losses (by parental education) within countries. Differences in the pandemic’s impacts on absolute mobility, therefore, largely reflect differences in the aggregate impact of school closures across countries.

In contrast to absolute mobility, the impact of the pandemic on relative mobility is driven entirely by the distribution of learning losses within countries. This is a consequence of unequal access to (or participation in) continued learning during school closures across socio-economic backgrounds, proxied by parental education. These impacts are significant given the historically slow-moving nature of this measure. They reverse some gains in relative mobility made by high-income countries since the generation of the 1970s and worsen an already declining trend in relative mobility in developing countries. A noteworthy feature of the results is that more optimistic assumptions about the effectiveness of alternative learning methods during school closures lead to more negative impacts on relative mobility. This reflects a key inequity: children from low-income families engaged less in any learning during school closures, so improvements in remote learning effectiveness disproportionally benefit children from better-off families.

The study’s findings are subject to several caveats. Simulations of long-term indicators such as social mobility are necessary as their evolution cannot be observed until well into the future, which would be too late to take mitigating policy steps. However, simulations require assumptions, including relying on the educational attainments of the 1980 cohort and estimated learning losses in LAYS, and assumed relative effectiveness of different remote learning modalities. Alternative modeling choices may lead to different results; our assumptions attempt to strike the right balance between theoretical defensibility and empirically feasibility for a sizeable number of developing countries.

A key assumption is that the 1980s cohort is a reasonable proxy for today’s cohort in terms of baseline intergenerational mobility. Yet, this may not hold. Educational attainment has generally increased overtime, albeit with a tapering gradient (Fig. 5), so both the pandemic-affected cohort and their parents are likely more educated than the 1980s cohort and their parents. This secular trend would not bias results if uniform across parent and child cohorts, or across socio-economic groups, but heterogeneity is likely. Tapering growth implies educational attainment may have increased faster for the parents of the pandemic cohort, than for the children, relative to the 1980s cohort. What would this imply for the analysis of absolute mobility? Consider the distance between children’s and parent’s years of schooling $d={Y}_{c}-{Y}_{p}$ and the probability of upward mobility as $P\left(d > 0\right)$. The higher gradient of educational improvement among parents implies a smaller average distance in the current cohort vis-à-vis the 1980s cohort. Simulating the loss of LAYS on the 1980s cohort would then understate the probability that children who would otherwise be upwardly mobile fail to surpass their parents after the shock. This implies that our estimates of the COVID-19 impacts on absolute mobility would be biased downward toward zero, and the true losses in absolute mobility would be even larger.

For relative mobility, Narayan et al. (2018) show that the correlation coefficient of parental and children’s education increased monotonically in developing countries between the 1960s and the 1980s cohorts. If this increase in persistence continued beyond the 1980s cohort (this is equivalent to assuming that increases in educational attainment were relatively more pronounced among children of higher socio-economic status relative, which seems plausible), then the simulations would be starting from a higher baseline (pre-COVID) covariance, if we had data for the current cohort. As the relative mobility measure is a function of this covariance between parental and children’s schooling, the lower persistence in the 1980s cohort would again imply that using the 1980s cohort would bias toward zero, or underestimate, the true impacts of the COVID-19 school closures on relative mobility.

A related concern is that identical LAYS losses can generate larger declines in absolute mobility—not necessarily because learning losses have a stronger causal effect, but due to ceiling effects or compositional differences in the parental education distribution across low- and high-income countries. Some of the simulations features mitigate these ceiling effect biases explicitly. The GDIM absolute mobility measure captures the share of a generation whose years of schooling exceed than the highest educational attainment among both parents, conditional on parents not having the maximum years of schooling observed in the sample, so that everyone has a chance of surpassing their parents. Furthermore, we simulate losses in educational attainment in % terms of pre-pandemic values, rather than in terms of absolute values, thus limiting floor effects at the lower end of the educational attainment. As the result, an equivalent LAYS loss will result in a relatively smaller simulated loss in educational attainment in low-income countries if the initial level of educational attainment is lower.

Note also that absolute mobility is lower for the 1940s–1980s cohorts in low-income countries, relative to middle- or high-income countries (Fig. 5 and Narayan et al. 2018). This suggests that the mechanical ease of surpassing parents’ schooling in low-income countries is not a first order concern; children in those countries continue to face significant difficulties in attaining high levels of education, for similar structural reasons. The main impacts of our simulations arise from longer periods of school closures and the higher quality of learning in middle- and high-income countries, which means that a given length of school closures leads to a larger loss in learning in terms of LAYS. Nevertheless, the absolute mobility simulations should be viewed in conjunction with the relative mobility simulations that do not suffer from the same ceiling constraints, and accounting for the cross-country heterogeneity that we report.

While we aimed for as comprehensive a coverage of developing country experience as the phone surveys would allow, we would caution against extrapolating too broadly from these results. Phone surveys were deployed in response to the cessation of regular data collection in the majority of countries during pandemic lockdowns, but HFPS data collection was also a function of local conditions, which varied considerably. Likewise, while HFPS data samples were drawn to be nationally representative (inclusively through post-survey adjustments), RDD sample designs exclude households without a telephone, who are more likely to be poorer, from the sampling frame (Brunckhorst et al. 2023b).

Finally, while we model differences in quality of instruction between the different learning modalities, there would also be quality differences within a given modality of learning, with higher-capacity countries being better able to deliver better quality instruction either through in-person interaction with teachers or through other remote learning modalities. We abstract from this heterogeneity, as it would be difficult to make reasonable assumptions on the quality of various modes of instruction across different countries.

For these reasons, the findings are best seen as illustrative and reflecting what might happen under the assumptions of the model, rather than being a statistical prediction of the future. Qualitative patterns across countries and comparisons with historical trends are more informative than precise numerical outputs, which could change as new data become available.

Furthermore, the COVID-19 pandemic not only interrupted schooling, but also reshaped household financial stability, educational aspirations and labor market conditions, with more pronounced impacts on financial stability, health and labor market outcomes of households at the lower end of the socio-economic spectrum (Bundervoet et al. 2023; Schady et al. 2023). Our simulations incorporate income effects to the extent that job and income losses affect the pandemic learning experience of children that we observe (Thomas et al. 2004; Oreopoulos et al. 2008). The simulations do not capture, however, any additional human capital impacts of closures due to schools being the conduits for other important programs, such as nutritional or early childhood interventions (Schady et al. 2023). These non-instructional interventions are often targeted toward vulnerable children, so abstracting from these likely makes our estimates conservative and thus a lower bound of the impact of school closures.

Interpreted thus, the results highlight serious implications for long-term poverty and inequality. Stalling or declining absolute mobility in education can contribute to intergenerational poverty traps since education is a primary ladder to upward mobility. Declining relative mobility, particularly where already low, represents worsening of inequality of opportunity that could lead to higher income inequality over time. Worsening of both types of mobility also implies a cost to potential long-term economic growth, as human potential is underutilized, and it may heighten risks to social stability in already strained societies.

Yet, these longer-term effects are not inevitable or irreversible. Learning losses need not become permanent losses in educational attainment if the right remedial measures are taken in time. A RAPID framework for learning recovery and acceleration has been introduced by UNICEF, UNESCO, USAID, the Bill and Melinda Gates Foundation, the UK FCDO and the World Bank, focusing on: (i) reaching every child and keeping them in school; (ii) assessing learning levels regularly; (iii) prioritizing teaching the fundamentals; (iv) increasing the effectiveness of instruction, including through catch-up learning; and (v) developing psychosocial health and well-being. This agenda aims to identify and re-enroll out-of-school children, inclusively through the expansion of second chance programs, deploy early warning systems for dropout risk, deploy national learning loss assessments and increase the capacity of teachers to deliver assessment-informed instruction, align curriculums to key skills and core content, provide targeted instruction and supplemental remediation to struggling students, as well as addressing the impacts of heightened stress and isolation (UNICEF 2023; World Bank 2023).

The time window is limited, however, and the more delayed these interventions are, the more costly and less effective they are likely to be to recover the learning losses. The urgency of addressing such longer-term risks can also sometimes get lost or de-emphasized in the face of pressing economic challenges whose effects are more apparent, which would be a mistake. Our simulation results hopefully will help strengthen the arguments for focusing on the human development impacts of the COVID-19 pandemic on children, even as other pressing short-term challenges are tackled; and also spur the development of policies that are able to minimize the education disruptions caused by future shocks including pandemics.

## Supplementary information

Supplementary_info_v3

## Data Availability

High Frequency Phone Survey data for most countries are available in a public, open-access repository available from the World Bank’s Microdata Library, High Frequency Phone Survey Catalog (https://microdata.worldbank.org/index.php/catalog/hfps).

## Footnotes

1 An important limitation of any measure of educational mobility, however, is that it does not reflect inequalities produced by the labor market (that affect income mobility) and are unrelated to differences in human capital, such as the extent to which access to good jobs may be influenced by the socioeconomic class one is born into (World Bank 2005; Narayan et al. 2018).

2 The 1980s cohort is the most recent cohort in GDIM for whom educational mobility could be accurately measured, given the dates of the underlying surveys and the fact that educational mobility can only be accurately measured for those who are old enough to have completed their education at the time of the survey.

3 In comparison, Neidhofer et al. (2021) and Neidhofer et al. (2022) do not have at their disposal any observational data on the education of children during the pandemic. Instead, they rely on national level information such as the duration of school closures, mitigation measures (e.g., availability of offline and online learning modalities) that were deployed, and group averages for indirect measures such as internet coverage among different socio-economic groups, as well as assumptions related to greater ability of parents with higher levels of education to substitute for lack of school instruction, to simulate the differences in learning losses of children for different types of households.

4 To see this, consider a scatterplot graph of parents’ and children’s years of education. A uniform loss due to the pandemic is equivalent to a parallel shift of the data points in this graph, which would not change the slope of the regression line.

5 More details on the International Standard Classification of Education (ISCED) can be found at: https://uis.unesco.org/en/topic/international-standard-classification-education-isced.

6 This is on account of a higher share of children whose parents have tertiary education reporting that they did not engage in any learning during the period of school closures, compared to households with primary or secondary education. One plausible reason for this unusual pattern could be schools in urban areas of Argentina (and thus with a greater share of more educated parents) having stricter lockdowns than schools in rural areas, resulting in fewer children in the latter group reporting no learning at all.
