# Supplementary material for: COVID-19 school closures, learning losses and intergenerational mobility
Source: Humanit Soc Sci Commun. 2026 Mar 21;13(1):646. doi: 10.1057/s41599-026-06967-w (PMC13171617; doi:10.1057/s41599-026-06967-w)
Supplement: Supplementary file 1 — Supplementary_info_v3 [file 41599_2026_6967_MOESM1_ESM.pdf]

Online Appendix:

COVID-19 School Closures, Learning Losses and Intergenerational Mobility

Annex 1:

COVID-19 Learning Loss simulations

The simulations of learning adjusted years of schooling losses relied upon in this analysis are described in detail in Azevedo et al. (2022). The main elements of the simulations are the following:

- **Learning gains** normally achieved during a regular school year before COVID. The higher the expected learning gains when schools are open, the higher the learning losses when schools close. These expected learning gains have a positive correlation with countries' income levels. The higher the income level of the country, the higher the expected learning gain. This parameter remains constant across scenarios (see Table A1.1 for a summary of the key input parameters on school closures, mitigation effectiveness, and school productivity (expected learning gain) used in the model to simulate the learning and earning outcomes under different scenarios).
- **Income shocks' impact on enrollments**. Simulations also partially capture the (much smaller) potential cumulative effects of household income shocks over the past two years on student school enrollment in primary education. This effect is negligible because evidence from both before and during COVID shows that at the primary-school level, income shocks typically have small effects on enrollment (Azevedo et al 2021; Evans and Moskovic 2022). This component varies across countries based on country-specific enrollment-income elasticities and growth projections and remain constant across scenarios.
- **Observed duration of school closures**, which ranged from a few weeks in some countries to nearly two years in others. We incorporate the latest country-specific school closure data, which covers two full years of schooling during COVID, from February 2020 to February 2022. As Figure A1.1. shows, there are significant differences in the school-opening policies of governments around the world. This component varies across countries and remain constant across scenarios.
- **Partial closure estimates**, the share of students in a school system who are assumed to be affected by partial closures. Partial closures can be by geographic location or by certain grades or can cover all students if a hybrid model is adopted. Very few countries have been able to monitor the share of their system partially closed. This parameter varies across scenarios.
- **Mitigation effectiveness:** mitigation strategies, particularly remote learning during school closures, are assumed, consistent with the observable evidence, typically not to have been effective. While some governments were able to respond swiftly to school closures by providing a variety of effective remote learning modalities, many were not. Most notably, 40 percent of countries in Sub-Saharan Africa did not provide any remote learning strategy despite full or partial school closures for about one year (Muñoz-Najar et al. 2021). Even in countries that did provide remote learning solutions, the provision of remote learning did not always result in take-up by students. Surveys of schools and households reveal that many children, especially in low-income countries, were not able to engage in remote learning at all (Meinck et al., 2022; UNESCO, UNICEF, World Bank, and OECD, 2021). Some countries experienced a "remote learning paradox" where the chosen remote learning approach was not suitable to the needs of the majority of the students, contributing to this uneven take-up (Muñoz-Najar et al. 2021). According to a survey of education ministries by UNESCO, UNICEF, World Bank and OECD (2021), over a third of low- and lower-middle income countries that provided lessons through radio or TV reported that less than half of primary school students were reached by these efforts. Even students who were able to receive some distance education often spent much less time learning than if they would have during in-person

instruction, and were exposed to pedagogies and curricula that had been hurriedly adapted to remote learning. Moreover, teachers often did not receive adequate training in remote instruction and digital skills (UNESCO, UNICEF, World Bank and OECD, 2021). Finally, as discussed below, data from actual measurement of learning losses support the assumption that mitigation was not effective: newly collected data on learning levels emerging from some low- and middle-income countries shows major learning losses across a range of contexts (UNESCO, UNICEF and World Bank, 2021).Building on the above-mentioned evidence and data from access to personal computers at home, Azevedo et al (2022) build a set of scenarios of mitigation effectiveness conditional on the different income levels of countries. These results yield a range of mitigation effectiveness in low- and middle-income countries from 5 to 20 percent of what an average student would learn while schools were open.

Figure A1.1: Extent of school closures and losses in LAYS

a. School closures and LAYS by region

b. School closures over time

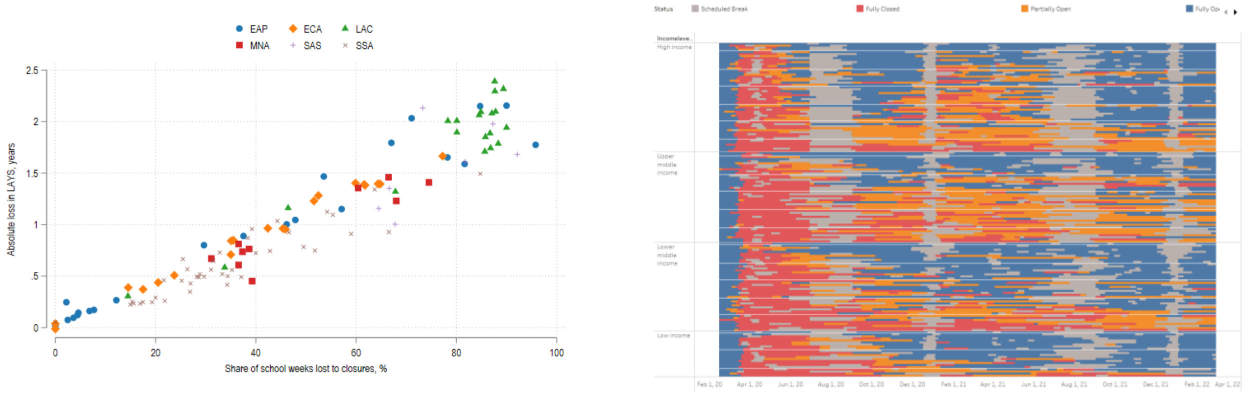

Source: Azevedo et al. (2022).

The overall learning losses expressed in LAYS can be decomposed into three different subcomponents, namely, the Expected Years of Schooling, the Harmonized Test Scores, and the losses due to dropouts. This decomposition shows that most of the simulated losses are channeled through the quantity and quality of education channels. A negligible amount is driven by the expected dropouts generated through the income shock (Figure A1.2.). This result is consistent with the emerging evidence arising from developed and developing countries that suggests that so far, it seems that the COVID-related school closures have not increased dropout rates.

Figure A1.2: Learning Losses simulations by different scenarios by LAYS components and main channels

(a) By income level

(b) By region (excluding High-income countries)

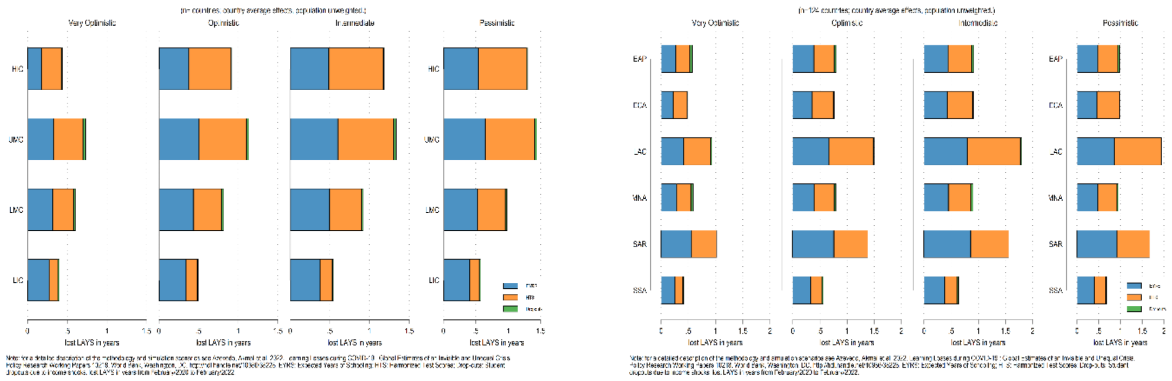

Source: Authors' calculations.

Table A1.1. Parameters for simulations by income level

	Global	High-Income	Upper-Middle-Income	Lower-Middle-Income	Low-Income
**A. Learning gains or school productivity (in HLO points/year)**	39	50	40	30	20
Optimistic Scenario					
B1. Share of the system affected over observed period (24 months)	42.9%	38.1%	52.7%	42.5%	34.4%
C1. Mitigation effectiveness (0 to 100%)	21.1%	30.0%	20.0%	14.0%	10.0%
D2. HLO decrease (points) = *B1*(A*((Total school weeks/43.3)*(1-C1))*	24.6	24.8	32.1	22.1	12.4
Intermediate Scenario					
B2. Share of the system affected over observed period (24 months)	45.4%	40.8%	55.9%	44.7%	36.0%
C2. Mitigation effectiveness (0 to 100%)	10.5%	15.0%	10.0%	7.0%	5.0%
D2. HLO decrease (points) = *B2*(A*((Total school weeks/43.3)*(1-C2))*	29.8	32.3	38.5	25.2	13.6
Pessimistic Scenario					
B3. Share of the system affected over observed period (24 months)	49.2%	44.8%	60.7%	48.0%	38.3%
C3. Mitigation effectiveness (0 to 100%)	10.5%	15.0%	10.0%	7.0%	5.0%
D3. HLO decrease (points) = *B3*(A*((Total school weeks/43.3)*(1-C3))*	32.3	35.4	41.7	27.0	14.5
**GEP* (GDP per capita growth %) [g]**	3.3	4	5.1	1.5	1.1

Note: Values represent country-level averages.

Notes: (*) Global Economic Prospects January 11th 2022 update (https://www.worldbank.org/en/publication/macro-poverty-outlook), with the regional average imputed if no country value was available for 2020 or 2021. The table provides an overview of parameters by country income level, though simulation parameters are applied at a country level. The World Bank's Harmonized Learning Outcome (HLO) puts learning data from international and regional assessments on a comparable scale. The data can be accessed here. We assume the learning gains will vary from 20 to 50 learning points depending on the country's income level, as explained in Azevedo, et al. (2021).

For illustrative purposes, in this study we focus on estimates of losses underlying the intermediate scenario in Azevedo et al. (2022), reflecting the evidence to date that mitigation strategies put in place have largely been ineffective; and many countries with educational systems that reported partial closures (on average for those last two years) had a large fraction of the system fully closed. The results in terms of global and regional effects on the LAYS do not vary dramatically across the scenarios.

Globally, the average baseline LAYS pre-COVID were 7.8, and 6.8 for low- and middle-income countries. This means that children around the world only achieve 7.8 years of quality education on average (compared to 11.9 non-quality adjusted years of schooling). The simulation results by Azevedo et al (2022) suggest that the average Learning Adjusted Years of School (LAYS) would fall due to COVID-19 school closures, bringing down the average learning that students achieve during their lifetime from 7.8 to 6.7 Learning Adjusted Years – a reduction of 1.1 years, as shown Table A1.2.

Across the globe, the extent of this loss varies. In Latin America and the Caribbean where children were expected to complete 7.8 years of LAYS prior to the pandemic, the simulations suggest that COVID-19 could lower LAYS by 1.5 years in the intermediate scenario (Table A1.2.). In South Asia, LAYS could fall from a baseline of 6.5, by 1.4 years in the intermediate scenario. At the other end of the spectrum, children in Sub-Saharan African were expected to complete 5 years of LAYS prior to COVID-19, and the simulations suggest that COVID-19 could lower LAYS by 0.6 years in the intermediate scenario. The variation in learning losses is related to differences in extent of school closures. In Latin America and the Caribbean, schools were fully closed for 225 days and partially closed for 236 days from February 2020 to February 2022. South Asia experienced 273 days of full closures and partial closures of 256 days. In contrast, Sub-Saharan Africa experienced relatively lower levels of school closures, with schools fully closed for 129 days and partially closed for 94 days on average.

These learning loss simulations are in the absence of learning recovery and acceleration in the post-pandemic years. The extent of learning recovery is yet unobserved, albeit emerging evidence suggests that learning recovery will be challenging. A recent study in the US estimates that for middle school cohorts it will take 5 years or more to recover the pandemic losses in reading and math, on average, and in high-poverty schools the time required for recovery will be even longer on account of larger learning losses during the pandemic (Kuhfeld and Lewis, 2022). Angrist et al. (2021) estimate that short-term learning losses due to COVID-19 for a child in grade 3 could accumulate to an equivalent 2.8 years of lost learning by grade 10. Schady et al. (2023) note that in Malawi learning trajectories were dramatically slower after seven months of school closures (6.9 points of learning for every 100 days of schooling upon reopening versus 13.4 points of learning for every 100 days of schooling pre-pandemic).

Learning losses can potentially be reversed over time, if appropriate recovery and acceleration programs are put in place. However, even before the pandemic the world was already experiencing a learning crisis (World Bank, 2018); and the latest learning poverty global updates showed that between 2015 and 2019, the world at best made no progress in reducing learning poverty (World Bank, UNESCO, UNICEF, FCDO, USAID, Gates Foundation, 2022). Learning recovery and acceleration is possible, but it requires strong political commitment and a broad national coalition bringing together government, teachers, parents and students with a clear focus on learning.

Successful experiences do exist. An after-school remedial program in Tamil Nadu that offered daily 60-90 minutes of remedial learning was estimated to have helped recover about half of the learning losses documented in December 2021 after a period of 6 months. The challenge, however, is often how to scale this intervention at the system level. Moreover, things can evolve in the opposite direction as well – evidence from Malawi suggests that not only there has been no catch-up learning, but the pace of learning post pandemic once schools reopened has been half as fast as it was before school closures, suggesting that the schools have not adjusted

successfully for effective recovery of learning losses (Asim, Gera and Singhal, 2022). An analysis of learning recovery by the World Bank in 2023, based on 60 countries spanning all developing country regions has found that only one in five countries had an explicit strategy in place to recovery and accelerate learning after school reopening; just 37 percent conducted assessments of the extent of learning losses (World Bank, 2023). Even in the United States, where the capacity of school systems to deploy resources towards learning recovery is much higher, data from 2024 show that 19 percent of states had a continued decline in proficiency rates since 2022 in English Language Arts (ELA), and a further 35 percent of states showed minimal change; in only 16 percent of states ELA proficiency levels in 2024 exceeded those recorded in 2019. For math, only 8 percent of states were above 2019 levels in 2024 (Kuhfeld and Lewis, 2025). Disaggregating the post-pandemic learning patterns by socioeconomic groups shows an even more sobering picture – according to the 2024 US National Assessment of Educational Progress (NAEP), there is a widening gap in performance between high performing and low performing students, with little recovery, or a continued decline among the latter – fourth and eighth graders in this group posted in 2024 the worst reading scores in over 30 years (NAEP, 2024).

Another prominent feature of the pandemic is the automatic re-enrollment and promotion that were one of the earliest recommendations made by several international organizations and adopted by most countries and were widely applied (UNICEF, 2020; ECLAC and UNESCO, 2020). A number of development agencies continue to recommend that countries legally mandate a policy of re-enrolling all students regardless of the duration of their absence (UNESCO, UNICEF, World Bank, and OECD, 2022). Such policy recommendations are likely to bias the expected years of schooling upward, at the same time as students experienced a reduction of their exposure to schooling and learning due to the COVID-related school closures. For instance, data from the latest Brazilian school census show a sharp increase in promotions, and a reduction in dropouts and repetitions across all grades during the pandemic, which have not yet returned to pre-pandemic levels. Rigotti et al (2013) show that the policies of automatic promotion between multi-year cycles of Brazilian states, such as São Paulo, had a significant impact on the value of expected years of schooling when adjusted by age. Thus, our simulations account for the actual losses of learning even when actual years of education completed may not differ.

Table A1.2. Effect on Learning Adjusted Years of Schooling (LAYS)

		Post-COVID 19			
	Baseline	Very Optimistic	Optimistic	Intermediate	Pessimistic
**Global**	7.8	7.2	6.9	6.7	6.7
**Global (Part 2)**	6.8	6.2	5.9	5.8	5.7
**By Region**					
East Asia and Pacific	8.3	7.8	7.6	7.4	7.4
Europe and Central Asia	10.0	9.6	9.3	9.1	9.0
Latin America and Caribbean	7.8	6.9	6.3	6.0	5.9
Middle East and North Africa	7.6	7.0	6.5	6.3	6.2
North America	11.1	10.5	9.6	9.1	8.8
South Asia	6.5	5.4	5.1	4.9	4.8
Sub-Saharan Africa	5.0	4.6	4.4	4.4	4.3
**By Region (Part 2)**					
East Asia and Pacific	7.3	6.7	6.5	6.4	6.3
Europe and Central Asia	8.9	8.5	8.2	8.0	8.0
Latin America and Caribbean	7.8	6.9	6.3	6.0	5.9
Middle East and North Africa	6.3	5.7	5.5	5.4	5.4
North America					
South Asia	6.5	5.4	5.1	4.9	4.8
Sub-Saharan Africa	5.0	4.6	4.4	4.4	4.3
**By income level**					
High Income	10.4	10.0	9.5	9.2	9.1
Upper middle income	7.8	7.1	6.7	6.5	6.4
Lower middle income	6.6	6.0	5.8	5.7	5.6
Low income	4.2	3.8	3.7	3.6	3.6
**By Lending type**					
Part 1	10.7	10.2	9.8	9.5	9.4
IBRD	8.0	7.3	6.9	6.7	6.6
IDA/Blend	5.7	5.2	5.0	4.9	4.9

Note: Results expressed in Learning-Adjusted Years of Schooling (LAYS) based on data for 174 countries (unweighted average). Calculations using the UNESCO School Closures database covering February 2020-February 2022 based on Azevedo et al., 2022; World Bank, UNESCO and UNICEF, 2021; and World Bank et al., 2022). For countries with learning data (LP, LAYS, or PISA) but no school closure data, we impute missing values for share of school system closed by using the regional average by income level. The estimates are country averages.

Annex 2:

Linking Expected Years of Schooling, Learning Adjusted Years of Schooling and Self-reported measures of educational attainment

Expected years of schooling (EYS) is a cohort measure, which is prospectively measured using a synthetic cohort. The actual years of schooling from age 7 to 14 can be observed and known; however, this procedure requires 7 years of observation over the span of the cohort's time in school. Alternatively, it is possible to carry out a retrospective reconstitution of this cohort, but data used to do so would be outdated for older individuals. The synthetic cohort borrows measurements of several real cohorts, observed during a period *t*, projected as though it would have been observed for ages at the respective times. Since period measurements can be taken in a single year and refer to data that are updated for that year, the synthetic cohort makes it possible to overcome the difficulties of real cohorts related to lengthy observation times and outdated data. EYS can also be calculated for periods as short as a single year (Figure A2.1).

Figure A2.1: Diagram Representing the Composition of Multicohorts, in the Period Measurement and in the Synthetic Cohort

Age											
14			8							8	
13			7						7		
12			6					6			
11			5				5				
10			4			4					
9			3		3						
8			2	2							
7			1								
	2020	2021	2022	2023	2024	2025	2026	2027	2028	2029	2030
	Year			Synthetic Cohort							

Source: Author's illustration.

In the steady state, and in the absence of any intervention and/or shocks, EYS and average educational attainment should be highly correlated, and stable. This can indeed be seen in Figure A2.2, which shows the correlation between EYS and self-reported years of schooling in GDIM data. The learning component of the LAYS comes from ratio (625/HTSc), where Harmonized Test Scores (HTS) for country c is a measure of quality of education, and is a population weighted average across all educational segments. Actual years of schooling in GDIM are, similarly, strongly correlated with LAYS.

Figure A2.2: GDIM years of schooling and expected years of schooling (left panel) and learning-adjusted years of schooling (right panel)

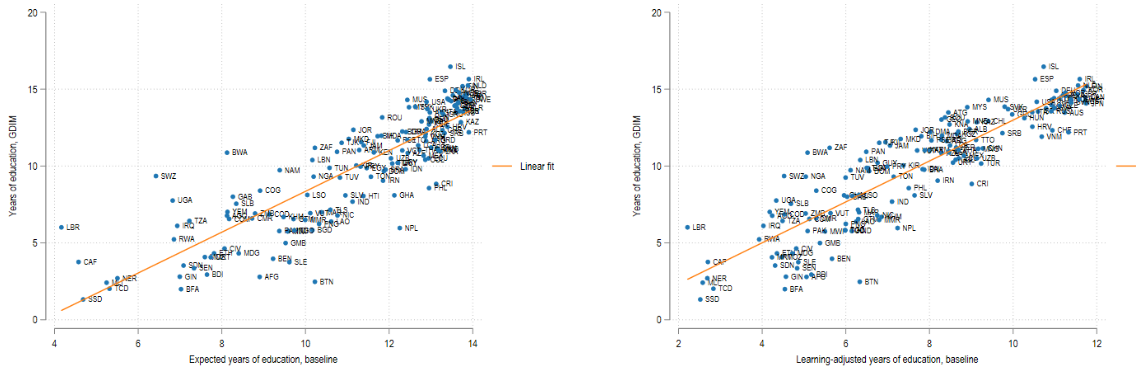

Source: Authors' estimates based on data from Azevedo et al. (2022) and GDIM.

Supplementary references

Angrist, Noam & de Barros, Andreas & Bhula, Radhika & Chakera, Shiraz & Cummiskey, Chris & DeStefano, Joseph & Floretta, John & Kaffenberger, Michelle & Piper, Benjamin & Stern, Jonathan, 2021. "Building back better to avert a learning catastrophe: Estimating learning loss from COVID-19 school shutdowns in Africa and facilitating short-term and long-term learning recovery," International Journal of Educational Development, Elsevier, vol. 84(C).

Asim, S., Gera, R., and Singhal, A. 2022. "Learning loss from Covid in Sub-Saharan Africa: Evidence from Malawi." Education for Global Development, April 19, 2022

Azevedo, João Pedro, Amer Hasan, Diana Goldemberg, Koen Geven, Syedah Aroob Iqbal. 2021. Simulating the Potential Impacts of COVID-19 School Closures on Schooling and Learning Outcomes: A Set of Global Estimates, The World Bank Research Observer, Volume 36, Issue 1, February 2021, Pages 1–40, https://doi.org/10.1093/wbro/lkab003

Azevedo, João Pedro; Akmal, Maryam; Cloutier, Marie-Helene; Rogers, Halsey; Wong, Yi Ning. 2022. Learning Losses during COVID-19 : Global Estimates of an Invisible and Unequal Crisis. Policy Research Working Papers; 10218. World Bank, Washington, DC.

ECLAC and UNESCO. 2020. Education in the time of COVID-19. Santiago de Chile: UNESCO, OREALC/CEPAL. August. https://unesdoc.unesco.org/ark:/48223/pf0000374075_eng

Kuhfeld, Megan, and Lewis, Karyn. 2022. "Student achievement in 2021-2022: Cause for hose and continued urgency." NWEA Research Brief, Portland, OR.

Kuhfeld, Megan, and Lewis, Karyn. 2025. "Five years after COVID-19 hit: Test data converge on math gains, stalled reading recovery", Brookings Institution, Washington, DC.

Meinck, S., Fraillon, J., & Strietholt, R. 2022. The impact of the COVID-19 pandemic on education: International evidence from the Responses to Educational Disruption Survey (REDS). UNESCO. https://unesdoc.unesco.org/ark:/48223/pf0000380398

Munoz-Najar, Alberto; Gilberto, Alison; Hasan, Amer; Cobo, Cristobal; Azevedo, Joao Pedro; Akmal, Maryam. 2021. Remote Learning During COVID-19 : Lessons from Today, Principles for Tomorrow. World Bank, Washington, DC

NAEP. 2024. "The Nation's Report Card: 2024 NAEP Reading Assessment" U.S. Department of Education, Washington, DC.

Rigotti, J.I.R., D.O. Sayyer, L. Rodriguez de Souza, C.G. Rodrigues. 2013. "A re-examination of the expected years of schooling: What can it tell us?" Working Paper No. 117, International Policy Centre for Inclusive Growth, UNDP, New York.

Schady, N., A. Holla, S. Sabarwal, J. Silva, and A. Y. Chang. 2023. *Collapse and Recovery: How the COVID-19 Pandemic Eroded Human Capital and What to Do about It*. Washington, DC: World Bank.

UNESCO; UNICEF; World Bank; OECD. 2021. What's Next? Lessons on Education Recovery: Findings from a Survey of Ministries of Education amid the COVID-19 Pandemic. UNESCO, Paris, UNICEF, New York, World Bank, Washington, DC, and OECD, Paris.

UNESCO, UNICEF, The World Bank and OECD (2022). From Learning Recovery to Education Transformation, Insights and Reflections from the 4th Survey of National Education Responses to COVID-19 School Closures. Montreal, New York, Washington D.C.: UNESCO-UIS, UNICEF, The World Bank and OECD.

UNICEF. 2020. Framework for reopening schools. New York: UNICEF. Retrieved from https://www.unicef.org/media/71446/file/Framework-for-reopening-schools.pdf

World Bank. 2018. *World Development Report 2018: Learning to Realize Education's Promise*. Washington, DC: World Bank.

World Bank. 2023. *Learning recovery to acceleration: A global update on country efforts to accelerate learning and reduce inequalities*. Washington, DC: The World Bank.

World Bank, UNESCO and UNICEF. 2021. The State of the Global Education Crisis: A Path to Recovery (English). Washington, D.C. : World Bank Group.

World Bank, UNESCO, UNICEF, USAID, FCDO and Gates Foundation. 2022. The State of Global Learning Poverty: 2022 Update (English). Washington, D.C.: World Bank Group.
